# Association between methylation of *BIN1* promoter in peripheral blood and preclinical Alzheimer’s disease

**DOI:** 10.1038/s41398-021-01218-9

**Published:** 2021-02-02

**Authors:** Hao Hu, Lan Tan, Yan-Lin Bi, Wei Xu, Lin Tan, Xue-Ning Shen, Xiao-He Hou, Ya-Hui Ma, Qiang Dong, Jin-Tai Yu

**Affiliations:** 1 Department of Neurology, Qingdao Municipal Hospital, Qingdao University, Qingdao, China; 2 Department of Anesthesiology, Qingdao Municipal Hospital, Qingdao University, Qingdao, China; 3 Department of Neurology and Institute of Neurology, WHO Collaborating Center for Research and Training in Neurosciences, Huashan Hospital, Shanghai Medical College, Fudan University, Shanghai, China

**Keywords:** Neuroscience, Clinical genetics

## Abstract

The bridging integrator 1 (*BIN1*) gene is the second most important susceptibility gene for late-onset Alzheimer’s disease (LOAD) after apolipoprotein E (*APOE*) gene. To explore whether the *BIN1* methylation in peripheral blood changed in the early stage of LOAD, we included 814 participants (484 cognitively normal participants [CN] and 330 participants with subjective cognitive decline [SCD]) from the Chinese Alzheimer’s Biomarker and LifestylE (CABLE) database. Then we tested associations of methylation of *BIN1* promoter in peripheral blood with the susceptibility for preclinical AD or early changes of cerebrospinal fluid (CSF) AD-related biomarkers. Results showed that SCD participants with significant AD biological characteristics had lower methylation levels of *BIN1* promoter, even after correcting for covariates. Hypomethylation of *BIN1* promoter were associated with decreased CSF Aβ42 (*p* = 0.0008), as well as increased p-tau/Aβ42 (*p* = 0.0001) and t-tau/Aβ42 (*p* < 0.0001) in total participants. Subgroup analysis showed that the above associations only remained in the SCD subgroup. In addition, hypomethylation of *BIN1* promoter was also accompanied by increased CSF p-tau (*p* = 0.0028) and t-tau (*p* = 0.0130) in the SCD subgroup, which was independent of CSF Aβ42. Finally, above associations were still significant after correcting single nucleotide polymorphic sites (SNPs) and interaction of *APOE* ɛ4 status. Our study is the first to find a robust association between hypomethylation of *BIN1* promoter in peripheral blood and preclinical AD. This provides new evidence for the involvement of *BIN1* in AD, and may contribute to the discovery of new therapeutic targets for AD.

## Introduction

Alzheimer’s disease (AD) is a complex, multifactorial neurodegenerative disease and is considered highly heritable^1^. Initial studies have identified some classical susceptibility genes, including amyloid precursor protein (*APP*), presenilin genes 1 and 2 (*PSEN1, PSEN2*) for early-onset AD (EOAD), and apolipoprotein E (*APOE*) for late-onset AD (LOAD). Subsequent genome-wide association studies identified a major susceptibility locus for LOAD on the bridging integrator 1 (*BIN1*) gene^2–4^, which was located on chromosome 2q14.3 and was currently identified as the second most important susceptibility gene in LOAD after *APOE*^5^. This association between genetic mutations in *BIN1* and AD susceptibility was also verified by our previous study on a large Han Chinese cohort^6^. As an important AD candidate gene, *BIN1* is widely expressed in many tissues and overexpressed *BIN1* has been observed in AD brains. Cellular, animal, and human studies have confirmed that BIN1 proteins have complex interactions with the pathological changes of AD^7–13^. However, genetic mutations alone do not seem to fully explain the abnormal expression of *BIN1* in patients with AD. Therefore, researchers have focused on epigenetic mechanisms.

The DNA methylation, as an important form of epigenetic mechanisms, has been suggested to be an important factor in the pathogenesis of AD^14^. Two large independent autopsy studies showed that there were methylation changes in the *BIN1* of the AD patient’s brain, accompanied by high expression of *BIN1*^15,16^. However, whether the *BIN1* methylation changes were also present in peripheral blood and whether they were associated with early pathology in LOAD patients were still unknown. In addition, our previous study has found that the expression of BIN1 protein in peripheral blood was significantly increased in LOAD patients and was negatively associated with the cognitive level^17^, which also aroused our interest in exploring the methylation status of *BIN1* in peripheral blood.

Recently, with the discovery of many biomarkers and the establishment of 2018 National Institute on Aging and Alzheimer’s Association (NIAA) research framework, studies of AD were more focused on the earlier stages, for example, the preclinical stage which was more important for the early prevention and intervention^18^. Subjective cognitive decline (SCD) in individuals without objective cognitive impairment has been suggested to be the first symptomatic expression of preclinical AD^19,20^. Therefore, based on a large population without objective cognitive impairment, consisting of completely cognitively normal (CN) and SCD individuals, this study aimed to explore associations of methylation of *BIN1* promoter in peripheral blood with the susceptibility for preclinical AD or early changes of cerebrospinal fluid (CSF) AD-related biomarkers. This may help to discover new pathogenic mechanisms or therapeutic targets for AD.

## Methods

### CABLE study

All participants without objective cognitive impairment in our study came from the Chinese Alzheimer’s Biomarker and LifestylE (CABLE) database. The CABLE database is an ongoing large independent cohort study initiated in 2017. It aimed to determine the genetic and environmental modifiers of AD biomarkers and their utility in early diagnosis in the northern Chinese Han population^21,22^. All participants in CABLE were enrolled at Qingdao Municipal Hospital, Shandong Province, China. The exclusion criteria include: (1) central nervous system infection, head trauma, neurodegenerative diseases other than AD (e.g., epilepsy, Parkinson’s Disease), or other major neurological disorders; (2) major psychological disorders; (3) severe systemic diseases (e.g., malignant tumors) that may affect CSF or blood levels of AD biomarkers including Aβ and tau; (4) family history of genetic diseases. All participants underwent clinical and neuropsychological assessments, biochemical tests, as well as blood and CSF sample collection. Comprehensive questionnaire, electronic medical record system, and a laboratory inspection management system were used to collect demographic information, AD risk factor profile, and medical history. Participants were aged between 40 and 90 years and consisted of individuals without objective cognitive impairment and individuals with MCI or AD. Each participant underwent comprehensive clinical, neuropsychological, psychosocial, and psychiatric evaluations to determine their cognitive diagnoses in compliance with the National Institute on Aging–Alzheimer’s Association (NIA-AA) workgroup diagnostic criteria^23–25^. Cognitive state of participants was tested using the China Modified Mini-Mental State Examination (CM-MMSE) and Montreal Cognitive Assessment (MoCA). Basic living ability was assessed by basic Activities of Daily Living score (ADL). Behavioral or psychological symptoms were assessed by Geriatric Depression Scale (GDS), Hamilton Rating Scale for Depression (HAMD), and Hamilton Rating Scale for Anxiety (HAMA). Vascular factors were assessed by Hachinski Inchemic Score (HIS).

The CABLE database was conducted in accordance with the Helsinki declaration, and the research program was approved by the Institutional Ethics Committee of Qingdao Municipal Hospital. Each participant signed an informed consent form.

### Study participants

This study included 814 participants who failed to meet the criteria of MCI and AD and were not been detected with objective cognitive impairment. The cognitive state of participants was tested by the CM-MMSE and MoCA. All participants had complete information including age, gender, years of education, CM-MMSE, *APOE* ε4 status, methylation levels of *BIN1* promoter, and levels of CSF AD core biomarkers including amyloid-β42 (Aβ42), total tau protein (t-tau), and phosphorylated tau protein (p-tau). The peripheral blood cell counts of each participant were also collected (neutrophile granulocyte, lymphocyte, monocyte, eosinophilic granulocyte, and basophilic granulocyte). The SCD was assessed by a Subjective Cognitive Decline Scale (SCDS) which was designed based on SCD-I recommendations^19,20^. We distinguished participants with SCD accompanied by particular concerns (worries) from those without objective cognitive impairment by the first section of SCDS which included a dichotomous question. Finally, we got 484 CN and 330 SCD participants. Detailed quality control information was shown in additional file 1 and the following sections.

### CSF sample collection and measurements

Fasting CSF samples was drawn through a standard operational process of lumbar puncture and processed within two hours after collection. Each specimen was centrifuged at 2000 × *g* for 10 min, and stored in an enzyme-free EP (Eppendorf) tube at −80 °C until subsequent assays. The thaw/freezing cycle did not exceed two times. CSF Aβ42, p-tau, and t-tau were determined with the ELISA kit (Innotest β-AMYLOID (1–42), hTAU-Ag, and PHOSPHO-TAU (181p); Fujirebio, Ghent, Belgium). The standards and CSF specimens were analyzed in duplicates, and the means values of the duplicates were used for subsequent statistical analyses. The inter-batch coefficient of variation (CV) was <20% (mean CV: 5.4% for Aβ42, 2.4% for p-tau, and 4.9% for t-tau). The intra-batch CV was <5% (mean CV: 4.5% for Aβ42, and 2.5% for p-tau, 4.4% for t-tau). All analyses were operated by professional experimenters who were blind to clinical information. Additional file 2 showed that CSF Aβ42 levels were reduced in *APOE* ε4 carriers and in older people. These results were consistent with those from previous studies^26^. In addition, there was no difference in inter-batch CV between CN and SCD subgroups (Additional file 1D–F).

Based on previous amyloid imaging^26,27^ and neuropathological studies^28–31^, approximately one-third of older adults without objective cognitive impairment had AD pathology in their brains. Therefore, in this study, CSF biomarker positive participants were defined as having CSF Aβ42 levels in the lower one-third of the distribution of participants (A+: ≤120.71 pg/mL) or having p-tau (T+: ≥39.15 pg/mL) or t-tau (*N*+: ≥184.19 pg/mL) levels in the upper one-third of the distribution. This method for establishing cut-offs was also used in previous studies, yielding reasonable results^22,32^.

### Blood sample collection and measurement

Blood samples of all the participants were drawn after at least a 12-h fasting period. QIAamp^®^ DNA Blood Mini Kit (250) was used to extract DNA from blood samples. And the extracted DNA was separated and stored in an enzyme free EP tube at −80 °C until *APOE* genotyping was completed in this study. Two specific loci (rs7412 and rs429358) of *APOE* were genotyped using the SNaPshot SNP assay. The peripheral blood cell composition was measured at the central laboratory of the Qingdao Municipal Hospital by an automated analytical platform (Sysmex XN-2800, Japan).

### The DNA methylation levels assay

The DNA methylation levels of CpG sites were determined by MethylTarget sequencing (Genesky Biotechnologies Inc., Shanghai, China), a method using next-generation sequencing-based multiple targeted CpG methylation analysis^33,34^. Primer design and validation were performed by Methylation Primer software on bisulfate-converted DNA. Six regions (BIN1_01–06) from CpG islands of *BIN1* gene were selected and sequenced (see additional file 3). Primer sets were designed to flank each targeted CpG site in 100–300 nucleotide regions. Genomic DNA was extracted from frozen samples using Genomic Tip-500 columns (Qiangen, Valencia, CA, USA) and bisulfate-converted using the EZ DNA Methylation™-GOLD Kit (Zymo Research, CA, USA) according to the manufacturer’s protocols. After PCR amplification (HotStarTaq polymerase kit, TAKARA, Tokyo, Japan) and library construction, samples were sequenced (Illumina HiSeq Benchtop Sequencer, CA, USA) using the paired-end sequencing protocol according to the manufacturer’s guidelines^35^. All analyses were operated by professional experimenters who were blind to clinical information.

In each sample, the percentage of sequences whose sequencing quality reaches Q30 value was higher than 90%. After calling methylation, we obtained the bisulfate conversion rate for each sample, and the samples with bisulfate conversion rate <99% were filtered out. After calculating the average coverage as well as the missing rate for each sample, the samples with average coverage <30-flod and/or with missing rate >0.01 were further filtered out. In our study, there was no difference in bisulfate conversion rate or sequence quality between CN and SCD subgroups (Additional file 1 B, C).

### Statistical analysis

The data were shown in the form of mean ± SD (standard deviation) or proportions of them. The outlier values which are situated outside three SD were excluded prior to subsequent analyses. The Shapiro–Wilk test was used to test normality for continuous variables. Mann–Whitney U test (for continuous variables) or Chisquare test (for categorical variables) were used for the comparison between groups. Pearson’s correlation coefficients were used to test the correlation between methylation levels of *BIN1* promoter and age. The area under the receiver operating characteristic curve (AUROC) was used to test the discriminatory efficacy of methylation levels. The methylation level of *BIN1* promoter was standardized by z-scale in logistic regression model to test its associations with preclinical AD susceptibility. All CSF variables were log10-transformed to ensure normality of data and were standardized by z-scale for comparison. Multiple linear regression models were used to test the associations between methylation levels of *BIN1* promoter and CSF biomarkers. All regression analyses were adjusted for age, gender, education, *APOE* ɛ4 status, and quality control variables including CV for CSF Aβ42, p-tau, and t-tau. Moreover, the interaction between *APOE* ɛ4 status and methylation levels were additionally added into multiple linear regression models when explored the influences of *APOE* ɛ4 status. The multicollinearity was assessed using tolerance, variance inflation factor (VIF), and Pearson’s correlation coefficients. No multicollinearity existed in each model of current study. Bonferroni correction was used for multiple comparisons. Statistical analyses were conducted using R, version 3.5.1. A two-tailed *p* < 0.05 was considered significant.

## Results

### Characteristics of participants

The participants’ characteristics were presented in Table 1. After strict quality control we analyzed data from 814 participants of the CABLE cohort, including 484 CN and 330 SCD participants. The average age of participants was 61.47 years; the average level of education was 9.91 years, 331 (40.66%) participants were female; and 122 (14.99%) participants were *APOE* ɛ4 carriers. No significant difference was found in gender distribution or level of education between two subgroups (*p* > 0.05). In comparison with CN subgroup, the SCD participants were older (*p* < 0.0001). As predicted, frequency of positive *APOE* ε4 status showed an increasing trend in SCD subgroup (CN: 13.84%, SCD: 16.67%), although not statistically significant.

**Table 1:** Characteristics of participants from CABLE database.

Variable	CN	SCD	Total	*p*
*N*	484	330	814	–
Age (year) mean (SD)	60.01 (10.47)	64.08 (9.88)	61.47 (10.06)	**<0.0001**^**#**^
Gender (Female/Male)	185/299	146/184	331/483	0.1002^£^
Education (year) mean (SD)	9.96 (4.43)	9.84 (4.38)	9.91 (4.41)	0.7892^#^
*APOE* ε4 carriers N (+/−, +/+)	67 (66, 1)	55 (50, 5)	122 (116, 6)	0.3134^£^
CM-MMSE mean (SD)	27.85 (2.36)	27.78 (2.21)	27.82 (2.29)	0.2533^#^
CSF Aβ42 (pg/mL) mean (SD)	195.79 (128.60)	168.19 (94.06)	184.60 (116.58)	**0.0004**^**#**^
CSF p-tau (pg/mL) mean (SD)	37.02 (9.74)	38.44 (11.12)	37.59 (10.34)	0.1727^#^
CSF t-tau (pg/mL) mean (SD)	172.40 (79.36)	187.34 (103.06)	178.46 (89.96)	0.0710^#^
CSF p-tau/Aβ42 mean (SD)	0.23 (0.10)	0.27 (0.12)	0.25 (0.11)	**<0.0001**^**#**^
CSF t-tau/Aβ42 mean (SD)	1.06 (0.63)	1.31 (0.92)	1.16 (0.77)	**<0.0001**^**#**^

*CN* cognitively normal participants, *SCD* participants with subjective cognitive decline, *APOE* apolipoprotein E gene, *CSF* cerebrospinal fluid, *Aβ* amyloid-β, *p-tau* phosphorylated tau protein, *t-tau* total tau protein, *CM-MMSE* China-Modified Mini-Mental State Examination, *SD* standard deviation.

Bold indicated that the results were statistically significant.

^#^Intergroup comparisons were tested by Mann–Whitney *U* test.

^£^Intergroup comparisons were tested by Chisquare test.

As for cognitive levels, we did not find significant cognitive difference between CN and SCD subgroups (CM-MMSE: *p* = 0.2533). As for CSF biomarkers, SCD subgroup had lower levels of Aβ42 (*p* = 0.0004) and higher levels of p-tau/Aβ42 (*p* < 0.0001) and t-tau/Aβ42 (*p* < 0.0001) compared with CN subgroup. No significant difference was found in levels of CSF p-tau (*p* = 0.1727) and t-tau (*p* = 0.0710) between two subgroups.

In addition, there was no significant difference in methylation level of *BIN1* promoter between different genders (*p* = 0.0741) and different *APOE* ɛ4 status (*p* = 0.2891), and no correlation was found between methylation levels of *BIN1* promoter and age (*p* = 0.4445).

### Methylation levels of *BIN1* promoter in different diagnostic groups

**Fig. 1:**
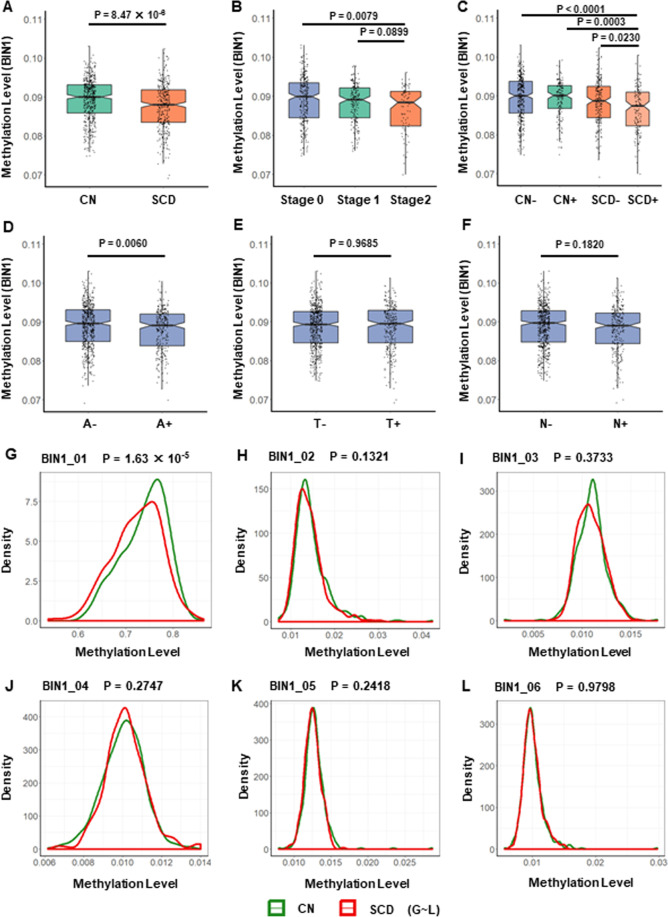
Methylation levels of *BIN1* promoter in different diagnostic groups. **A** Methylation levels of *BIN1* promoter in a clinical diagnostic construct. CN (*N* = 484): cognitively normal individuals; SCD (*N* = 330): individuals with subjective cognitive decline; **B** methylation levels of *BIN1* promoter in an ATN biological construct. Stage 0: A−T−N− (*N* = 290), Stage 1: A+T−N− (*N* = 189), Stage 2: A+T+N− (*N* = 12), A+T−N+ (*N* = 18), A+T+N+ (*N* = 54); **C** methylation levels of *BIN1* promoter in a diagnostic structure combining clinical diagnosis and biomarkers. CN− (*N* = 343): cognitively normal individuals with negative CSF Aβ42 [A-]; CN+(N = 141): cognitively normal individuals with positive CSF Aβ42 [A+]; SCD− (*N* = 194): individuals with subjective cognitive decline and negative CSF Aβ42 [A−]; SCD+ (*N* = 136): individuals with subjective cognitive decline and positive CSF Aβ42 [A+]. **D**–**F** Methylation levels of *BIN1* promoter in A or T or N construct, respectively. **G**–**L** Methylation levels of different regions on *BIN1* promoter. BIN1 bridging integrator 1 gene promoter, BIN1_01–06 six different regions of bridging integrator 1 gene promoter. All intergroup comparisons were tested by Kruskall–Wallis test and Wilcoxon test.

In a clinical diagnostic construct (Fig. 1A), SCD subgroup showed significantly lower methylation levels of *BIN1* promoter compared with CN subgroup (*p* = 8.47 × 10^−6^). In an ATN biological construct, the A+ subgroup had significantly lower methylation levels of *BIN1* promoter than the A− subgroup (*p* = 0.0060) (Fig. 1D–F). Then, according to the pathological changes in the ATN framework, we resulted three different biomarker group combinations including stage 0, stage 1, stage 2 (Fig. 1B). Specifically, participants with three negative CSF biomarkers (A−T−N−) were classified as the stage 0 subgroup. Participants with positive Aβ42 as well as negative p-tau and t-tau (A+T−N−) were classified as the stage 1 subgroup. Participants with positive Aβ42 as well as positive p-tau or t-tau (A+T+N−, A+T−N+, A+T+N+) were classified as the stage 2 subgroup. The methylation levels of *BIN1* promoter showed a gradually decreasing trend from stage 0 to stage 2. The stage 2 subgroup had significantly lower methylation levels than the stage 0 subgroup (*p* = 0.0079). Furthermore, in a diagnostic structure combining clinical diagnosis and biomarkers (Fig. 1C), SCD participants with A+(SCD+) had much lower methylation levels of *BIN1* promoter than CN participants without significant Aβ42 pathological changes (CN−) (*p* < 0.0001).

Further analysis of different *BIN1* promoter regions showed that the above differential methylation levels between CN and SCD subgroups only existed in the BIN1_01 region (*p* = 1.63 × 10^−5^), but not in other regions (BIN1_02–06) (Fig. 1G–L). In support of this result, some nearby differential methylation CpG sites were found in BIN1_01 region (Additional file 4).

The above analyses found differential methylation levels of *BIN1* promoter between different diagnostic groups. However, we could also see that these methylation levels in different diagnostic groups showed a high degree of overlap, which indicated a low discriminatory efficacy (AUROC [CN vs. SCD]: 0.5918; AUROC [CN− vs. SCD+]: 0.6448) (Additional file 5).

### The results of logistic regression analysis

**Fig. 2:**
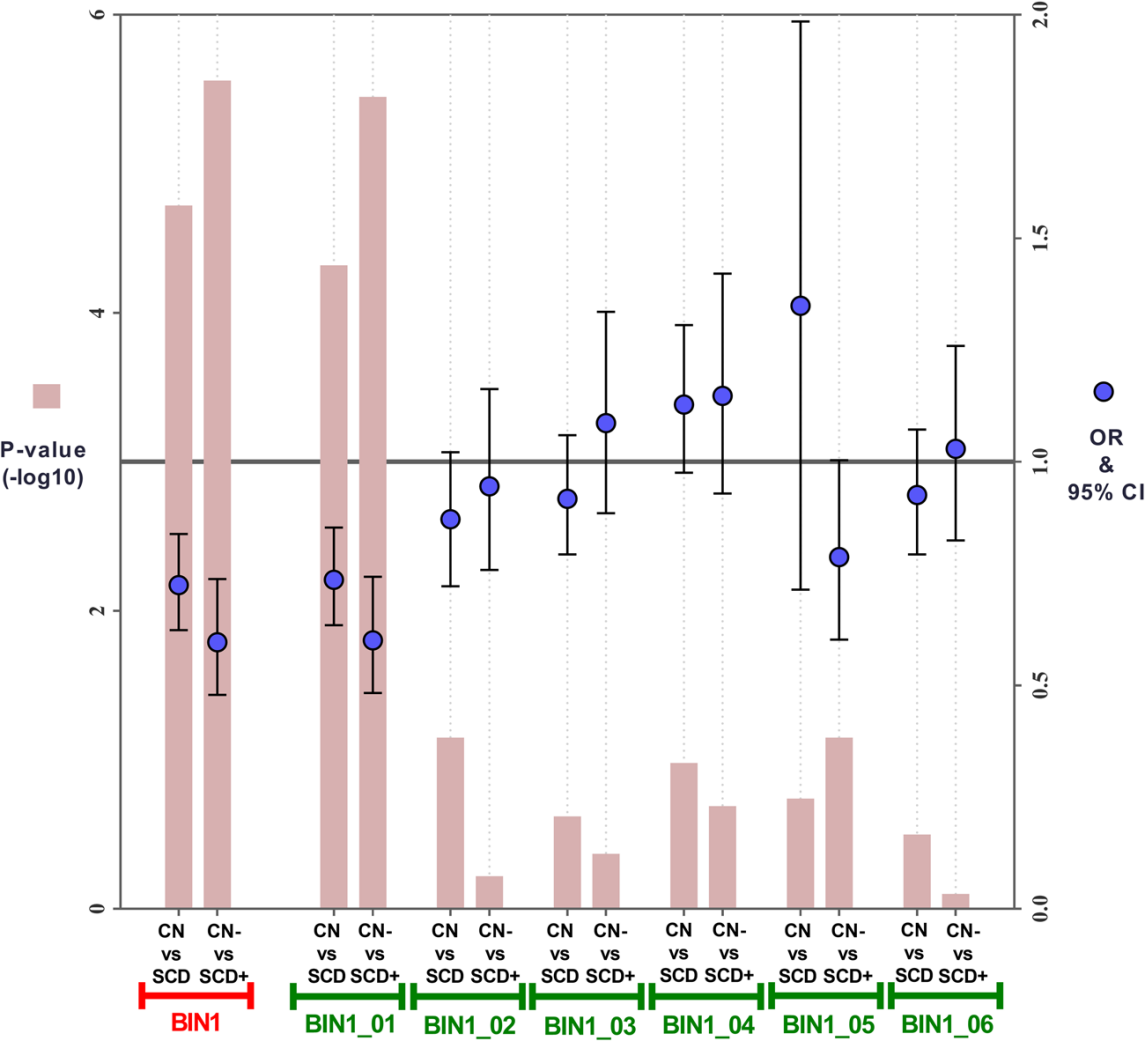
The results of logistic regression analysis. Abbreviations: CN cognitively normal participants, SCD participants with subjective cognitive decline, CN− cognitively normal participants with negative CSF Aβ42 [A−], SCD+ participants with subjective cognitive decline and positive CSF Aβ42 [A+], BIN1 bridging integrator 1 gene promoter; BIN1_01–06 six different regions of bridging integrator 1 gene promoter, OR odds ratio, CI confidence interval. Logistic regression models were used to test the associations between methylation levels of *BIN1* promoter and preclinical AD susceptibility, adjusting for age, gender, education, and *APOE* ɛ4 status.

Then, after adjusting for age, gender, education, and *APOE*ɛ4 status, we further tested the associations between methylation levels of *BIN1* promoter and preclinical AD susceptibility in logistic regression models. Results still showed that lower methylation levels of *BIN1* promoter were associated with SCD or SCD+ (*p* < 0.05) (Fig. 2).

### Associations between methylation levels of *BIN1* promoter and CSF AD core biomarkers in total participants

Table 2 shows the results on associations between methylation levels of *BIN1* promoter and CSF AD core biomarkers in total participants. Lower methylation levels of *BIN1* promoter were significantly associated with decreased levels of CSF Aβ42 (*β* = 20.1268, *p* = 0.0008) as well as increased levels of CSF p-tau/Aβ42 (*β* = −23.8708, *p* = 0.0001) and t-tau/Aβ42 (*β* = −24.5219, *p* < 0.0001). No association of methylation levels was found with t-tau or p-tau.

**Table 2:** Associations between methylation levels of *BIN1* promoter and CSF biomarkers in total and different diagnostic subgroups.

Variable	Total		CN		SCD	
	*β*	*p*	*β*	*p*	*β*	*p*
*BIN1*						
CSF Aβ42	20.1268	**0.0008**	9.5133	0.2527	30.1634	**0.0006**
CSF p-tau	−6.6890	0.2600	12.8908	0.1118	−26.9626	**0.0028**
CSF t-tau	−8.5145	0.1446	5.6175	0.4820	−22.1300	**0.0130**
CSF p-tau/Aβ42	−23.8708	**0.0001**	−4.4539	0.5910	−43.3853	**<0.0001**
CSF t-tau/Aβ42	−24.5219	**<0.0001**	−4.2688	0.6043	−41.8846	**<0.0001**
*BIN1_01*						
CSF Aβ42	2.2962	**0.0006**	1.1389	0.2169	3.4004	**0.0005**
CSF p-tau	−0.8155	0.2190	1.3984	0.1200	−3.1768	**0.0017**
CSF t-tau	−0.9745	0.1347	0.6449	0.4669	−2.5874	**0.0096**
CSF p-tau/Aβ42	−2.7395	**<0.0001**	−0.5862	0.5237	−4.9754	**<0.0001**
CSF t-tau/Aβ42	−2.7966	**<0.0001**	−0.5222	0.5676	−4.8110	**<0.0001**

Bold indicated that the results were statistically significant.

Multiple linear regression models were used to test the associations between methylation levels of BIN1 promoter and CSF biomarkers, adjusting for age, gender, education, and *APOE* ɛ4 status.

*CN* cognitively normal participants, *SCD* participants with subjective cognitive decline, *CSF* cerebrospinal fluid, *Aβ* amyloid-β, *p-tau* phosphorylated tau protein, *t-tau* total tau protein, *BIN1* bridging integrator 1 gene promoter, *BIN1_01* 01 region of bridging integrator 1 gene promoter.

As for different regions on *BIN1* promoter, the above associations only existed in the BIN1_01 region but not other five regions (BIN1_02–06) (Additional file 6).

### Associations between methylation levels of *BIN1* promoter and CSF AD core biomarkers in different diagnostic subgroups

In the subgroup analysis of different diagnostic groups, those associations between methylation levels of *BIN1* promoter and Aβ-related biomarkers still remained significant in SCD subgroup (Aβ42, *p* = 0.0006; p-tau/Aβ42, *p* < 0.0001; t-tau/Aβ42, *p* < 0.0001) but not in CN subgroup. In addition, lower methylation levels of *BIN1* promoter were also associated with increased CSF p-tau (*β* = −26.9626, *p* = 0.0028) and t-tau (*β* = −22.1300, *p* = 0.0130) in SCD subgroup, while these associations were not found in CN subgroup (Table 2).

**Fig. 3:**
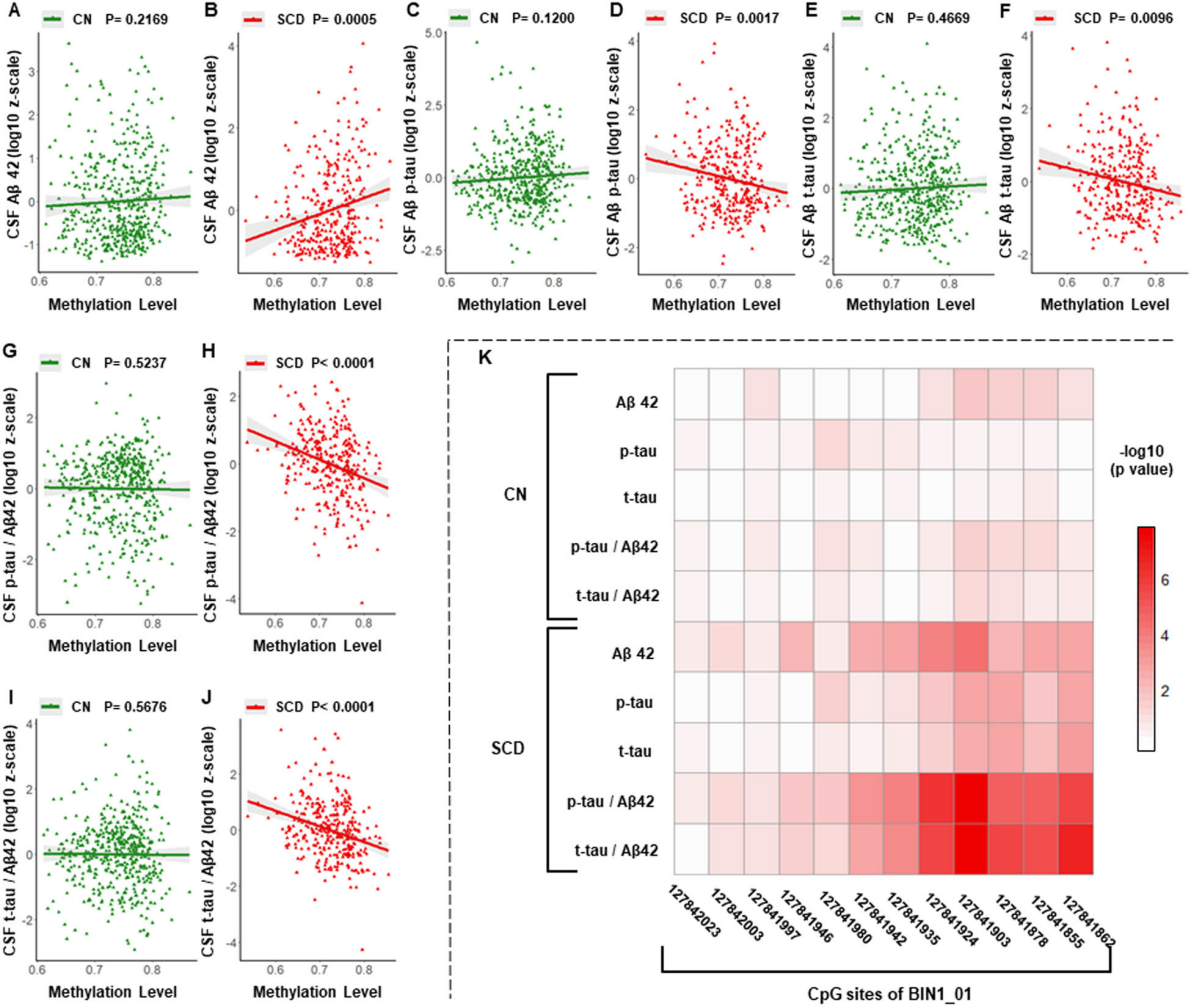
Associations between methylation levels of *BIN1* promoter and CSF AD biomarkers in different diagnostic subgroups. Abbreviations: CN cognitively normal participants, SCD participants with subjective cognitive decline, CSF cerebrospinal fluid, Aβ amyloid-β, p-tau phosphorylated tau protein, t-tau total tau protein, BIN1_01 the 01 region of bridging integrator 1 gene promoter. **A**–**J** Methylation levels of BIN_01 region on *BIN1* promoter were associated with CSF AD biomarkers in SCD subgroup, but not in CN subgroup. **K** Methylation levels of some nearby CpG sites on BIN1_01 region were associated with CSF AD biomarkers in SCD subgroup, but not in CN subgroup. Multiple linear regression models were used to examine the associations between methylation levels of *BIN1* promoter and CSF biomarkers, adjusting for age, gender, education and *APOE* ɛ4 status.

Similarly, as for different regions on *BIN1* promoter, these associations found in the SCD subgroup only existed in the BIN1_01 region (Table 2, Fig. 3A–J) but not in other five regions (BIN1_02–06) (Additional file 6). In addition, on BIN1_01 region, methylation levels of some nearby CpG sites, which have been found to be associated with the preclinical AD susceptibility in above analyses, were also found to be associated with CSF AD core biomarkers. Those associations were much more significant in SCD subgroup (Fig. 3K).

### Mediation analyses

Furthermore, we explored the potential mediation role of CSF Aβ42 in the associations between methylation levels of *BIN1* promoter and tau-related biomarkers (CSF p-tau or t-tau) in SCD subgroup. To do so, we repeated the analysis of tau-related biomarkers by further controlling for CSF Aβ42 changes. Results showed that those methylation associations with tau-related biomarkers remained significant after controlling for Aβ42 (*p* < 0.05).

### Influences of genetic factors on associations between methylation levels of *BIN1* promoter and preclinical AD susceptibility or pathological changes

**Fig. 4:**
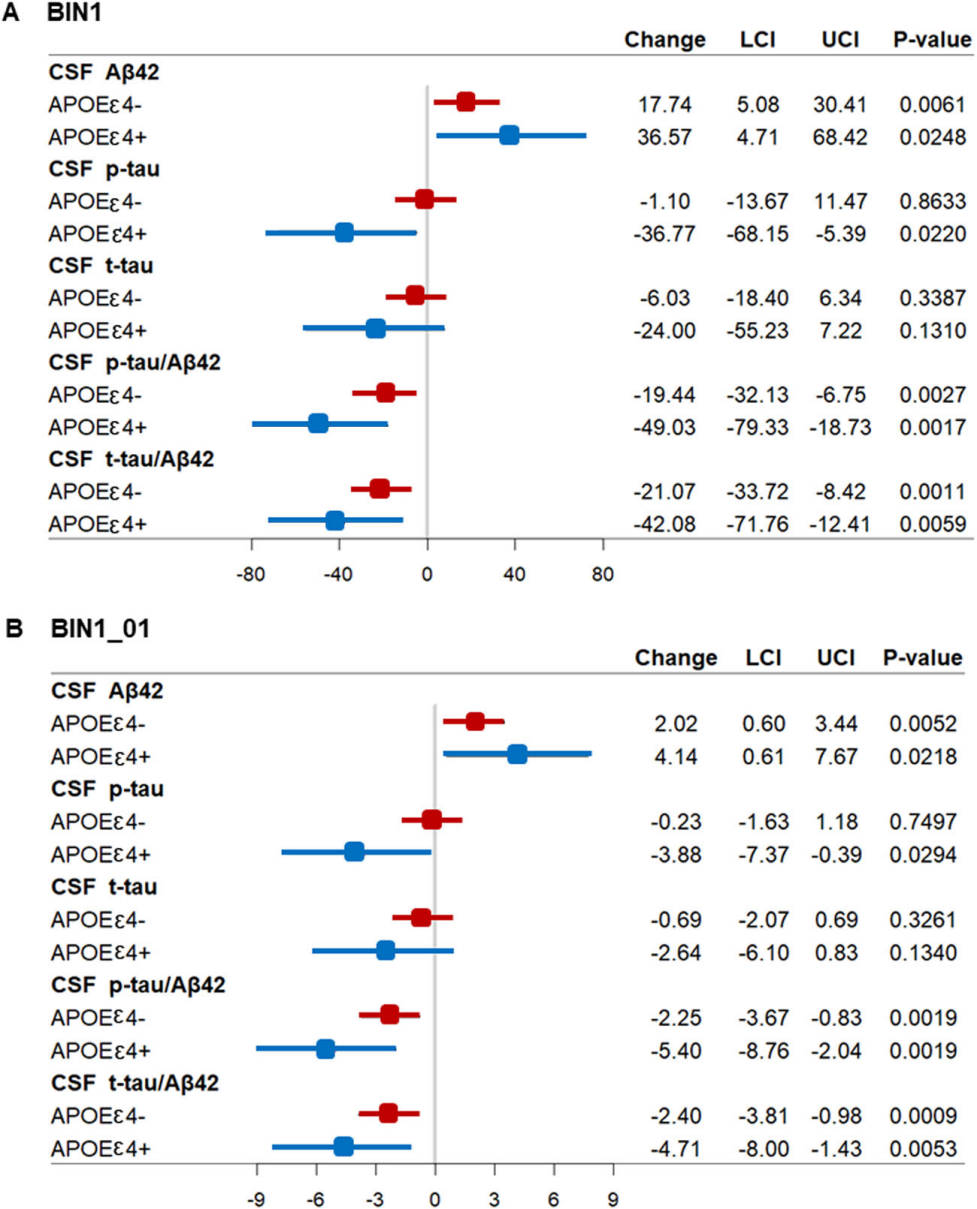
Associations between methylation levels of *BIN1* promoter and CSF AD biomarkers in different *APOE* ε4 status. Abbreviations: CSF cerebrospinal fluid, Aβ amyloid-β, p-tau phosphorylated tau protein, t-tau total tau protein, *APOE* apolipoprotein E gene, LCI low value of 95% confidence interval, UCI up value of 95% confidence interval, BIN1 bridging integrator 1 gene promoter, BIN1_01 the 01 region of bridging integrator 1 gene promoter. **A** Associations between methylation levels of *BIN1* promoter and CSF AD biomarkers in different *APOE* ε4 status. **B** Associations between methylation levels of BIN1_01 region on *BIN1* promoter and CSF AD biomarkers in different *APOE* ε4 status. Multiple linear regression models were used to examine the associations between methylation levels of *BIN1* promoter and CSF biomarkers, adjusting for age, gender, and education.

To clarify the influence of *APOE* gene on the above associations, we performed two analyses. Firstly, we added the interaction between *APOE* ɛ4 status and methylation levels into multiple linear regression models. Results showed that methylation levels of *BIN1* promoter were still significantly associated with CSF Aβ42, p-tau/Aβ42, and t-tau/Aβ42 in these models. Though the interaction (BIN1×*APOE*) was not significantly associated with the changes of CSF Aβ42, t-tau, and t-tau/Aβ42 (*p* > 0.01), it had moderate influences on the level of CSF p-tau (*p* = 0.0195) and p-tau/Aβ42 (*p* = 0.0857) (Table 3). Secondly, the subgroup analysis of *APOE* ɛ4 status showed that associations of methylation levels with CSF Aβ42, p-tau/Aβ42, and t-tau/Aβ42 were significant for both *APOE* ɛ4 carriers and non-carriers (Fig. 4). Interestingly, as shown in Fig. 4, the effects of methylation levels on these three CSF biomarkers (Aβ42, p-tau/Aβ42, t-tau/Aβ42) seemed to be more obvious in *APOE* ɛ4 carriers, though these differences did not reach statistical significance in above interaction analysis probably because of the limited number of *APOE* ɛ4 carriers. Moreover, a negative association of methylation levels with CSF p-tau was found only in the *APOE* ɛ4 carriers but not non-carriers.

**Table 3:** Associations between methylation levels of *BIN1* promoter and CSF biomarkers after adjusting interactions between methylation and *APOE* ε4 status.

Variable	Model1 (BIN1)				Model2 (BIN1_01)			
	BIN1		BIN1×*APOE*		BIN1_01		BIN1_01×*APOE*	
	*β*	*p*	*β*	*p*	*β*	*p*	*β*	*p*
CSF Aβ42	18.5643	**0.0038**	10.9614	0.5008	2.1285	**0.0029**	10.6084	0.5142
CSF p-tau	−1.2903	0.8391	−37.9497	**0.0195**	−0.2277	0.7479	−37.2680	**0.0216**
CSF t-tau	−5.7126	0.3614	−19.6861	0.2176	−0.6670	0.3394	−19.4800	0.2220
CSF p-tau/Aβ42	−19.8966	**0.0018**	−27.8422	0.0857	−2.3084	**0.0011**	−27.2482	0.0921
CSF t-tau/Aβ42	−20.9574	**0.0009**	−24.9688	0.1202	−2.4083	**0.0006**	−24.5243	0.1265

Bold indicated that the results were statistically significant.

Multiple linear regression models were used to test the associations between methylation levels of *BIN1* promoter and CSF biomarkers, adjusting for age, gender, education, *APOE* ɛ4 status, and interaction between methylation and *APOE* ε4 status.

*CSF* cerebrospinal fluid, *Aβ* amyloid-β, *p-tau* phosphorylated tau protein, *t-tau* total tau protein, *APOE* apolipoprotein E gene, *BIN1* bridging integrator 1 gene promoter, *BIN1_01* 01 region of bridging integrator 1 gene promoter.

In addition to the *APOE* gene, some single nucleotide polymorphic sites (SNPs) in *BIN1* promoter may also affect the above associations. Therefore, we screened out two SNPs whose minor allele frequency was higher than 0.05 (rs58402148: 0.13; rs17014923: 0.10). Though we did not find their associations with preclinical AD susceptibility and CSF AD core biomarkers (*p* > 0.05), they showed significant associations with methylation levels of *BIN1* promoter (*p* < 0.0001) (Additional file 7). Then we added those two SNPs into regression models. Results showed that the associations of methylation levels of *BIN1* promoter with preclinical AD susceptibility (CN vs. SCD: *p* = 0.0002; CN− vs. SCD+: *p* < 0.0001) or CSF biomarkers changes (Table 4) remained significant.

**Table 4:** Associations between methylation levels of *BIN1* promoter and CSF biomarkers after adjusting SNPs.

Variable	Total		CN		SCD	
	*β*	*p*	*β*	*p*	*β*	*p*
*BIN1*						
CSF Aβ42	28.9148	**0.0001**	15.3313	0.1312	45.5261	**<0.0001**
CSF p-tau	−13.2467	0.0708	1.9133	0.8457	−29.2024	**0.0103**
CSF t-tau	−11.5700	0.1090	4.4243	0.6501	−27.7005	**0.0143**
CSF p-tau/Aβ42	−36.2108	**<0.0001**	−15.9422	0.1145	−59.0300	**<0.0001**
CSF t-tau/Aβ42	−34.9525	**<0.0001**	−11.6603	0.2461	−58.1587	**<0.0001**
*BIN1_01*						
CSF Aβ42	3.2062	**0.0001**	1.7765	0.1099	4.9218	**0.0001**
CSF p-tau	−1.5151	0.0600	0.2198	0.8382	−3.4143	**0.0062**
CSF t-tau	−1.2881	0.1050	0.5215	0.6250	−3.1605	**0.0107**
CSF p-tau/Aβ42	−4.0228	**<0.0001**	−1.8270	0.0983	−6.5380	**<0.0001**
CSF t-tau/Aβ42	−3.8733	**<0.0001**	−1.3162	0.2314	−6.4468	**<0.0001**

Bold indicated that the results were statistically significant.

Multiple linear regression models were used to test the associations between methylation levels of BIN1 promoter and CSF biomarkers, adjusting for age, gender, education, *APOE* ɛ4 status and two SNPs (rs58402148; rs17014923).

*CN* cognitively normal participants, *SCD* participants with subjective cognitive decline, *CSF* cerebrospinal fluid, *Aβ* amyloid-β, *p-tau* phosphorylated tau protein, *t-tau* total tau protein, *BIN1* bridging integrator 1 gene promoter, *BIN1_01* 01 region of bridging integrator 1 gene promoter.

### Sensitivity analyses

Given that peripheral blood cell composition and storage time of samples might affect methylation differences between individuals, we further recalculated the above results after additionally adjusting peripheral blood cell composition (neutrophile granulocyte, lymphocyte, monocyte, eosinophilic granulocyte, and basophilic granulocyte) and storage time of samples. These results did not change significantly (Additional file 8).

## Discussion

This study was the first to systematically explore the associations of methylation of *BIN1* promoter in peripheral blood with preclinical AD susceptibility and early pathological changes of CSF AD core biomarkers in a large cohort of participants without objective cognitive impairment. The main finding of our study was that hypomethylation of *BIN1* promoter might increase the risk of preclinical AD and be associated with more severe pathological changes of CSF AD core biomarkers in elderly adults without objective cognitive impairment. This finding was novel and potentially important. It provided new evidence for the involvement of *BIN1* gene in the pathogenesis of AD, suggesting *BIN1* may be a new therapeutic target for the treatment of AD.

Firstly, consistent with previous studies^36,37^, our analysis of the participant characteristics showed that though the SCD participants in our study did not have an objective cognitive impairment, they had obvious changes in CSF AD core biomarkers (especially Aβ-related biomarkers). These results suggested that, in our study, SCD participants had preclinical AD characteristics^20^.

Then we found that SCD participants, especially the SCD participants with obvious Aβ-related pathologic changes, had lower methylation levels of *BIN1* promoter compared with controls, which indicated that hypomethylation of *BIN1* promoter might increase the risk of preclinical AD. Consistent with our results, a recent study of differential methylation in peripheral blood between cognitively normal people and patients with AD or MCI found some differentially methylated loci near *BIN1* gene^38^. Moreover, two large independent autopsy studies on human brain tissues, published separately in the *Nature Neuroscience* and *JAMA Neurology*, found that methylation in *BIN1* gene was associated with pathologic diagnosis of AD, even in individuals without objective cognitive impairment^15,16^. Therefore, all these evidences in both human peripheral blood and brain tissues showed that methylation in *BIN1* gene was associated with the susceptibility of preclinical AD.

Generally speaking, altered DNA methylation (especially in promoters) may lead to changes in gene expression. In fact, as for human brain tissue, both *BIN1* mRNA^15,16,39,40^ and protein^40,41^ levels were altered in AD patients. As for human peripheral blood, our previous study in a northern Han Chinese population observed a marked increase in *BIN1* mRNA and protein levels in AD patients, as well as a strong negative association between BIN1 protein levels and cognitive level^17^. Overall, both brain and peripheral blood evidences suggested that the expression of the *BIN1* gene changed, indirectly supporting hypothesis that altered methylation of *BIN1* may contribute to the occurrence and development of AD by regulating the expression of RNA or protein.

To further clarify the association between the methylation status of *BIN1* promoter in peripheral blood and preclinical AD, the associations between methylation of *BIN1* promoter and early pathological changes were explored in participants, especially in SCD individuals. We found that hypomethylation of *BIN1* promoter was associated with early pathological changes in preclinical AD, which is consistent with previous studies on brain tissue showing that methylation at some CpG sites in *BIN1* was associated with Aβ load and tau tangle density^15,16^. In addition, studies on the interaction between BIN1 protein and AD pathology also supported the involvement of *BIN1* in early pathological changes of AD. Specifically, as for Aβ-related pathology, BIN1 protein was thought to be involved in endocytosis, which could serve as a pathway that leads to APP production and release^7,8^. Super-resolution microscopy and immunogold electron microscopy analyses highlighted the presence of BIN1 protein in proximity to amyloid fibrils at the edges of amyloid deposits, which revealed that the aberrant accumulation of BIN1 protein was a feature associated with AD amyloid pathology^10^. As for tau-related pathology, besides affecting the endocytosis of tau protein^9^, BIN1 protein can also directly bind to a proline-rich domain in tau by SH3 domain, suggesting that increased expression of BIN1 protein exacerbates tau-related pathology^11,13^. Moreover, BIN1 protein was also directly involved in modulating tau-related actin dynamics^12^. Overall, from the above studies, it was not difficult to see that BIN1 protein was involved in the pathological changes of AD and the mechanisms of its influences on Aβ and tau pathology were significantly different. Consistent with these differences in mechanisms, results in our study and previous brain tissue studies^16^ all showed that the associations between methylation status of *BIN1* and tau pathology was not completely dependent on Aβ pathology. In other words, these results suggested that methylation of *BIN1* might have independent effects on these two molecular processes.

From the above analyses, we have got significant associations of *BIN1* promoter methylation status and susceptibility with preclinical AD or early pathological changes of CSF AD biomarkers. However, it was important to note that a variety of other genetic factors may affect or mediate those associations. For example, some SNPs in *BIN1* have been reported to show replicable associations with the susceptibility^2–4^ or biomarkers^42^ of LOAD in different independent populations, including a large northern Han Chinese population^6^. Therefore, we explored the influences of SNPs and *APOE* ɛ4 status (the primary risk gene of LOAD) on those associations. Results showed that those associations in peripheral blood were not entirely dependent on genetic factors. This independence was also seen in several previous studies on human brain tissue^15^. In addition, some studies have shown the presence of tissue or cell heterogeneities in the expression of *BIN1*^40,43^. Such heterogeneities in expression also indirectly suggested the presence of epigenetic regulation rather than simple genetic mutations, due to the plasticity of the epigenome. However, it was worth noting that a modest effect of interaction between *APOE* ɛ4 status and methylation levels was also found in our study, especially for the CSF p-tau levels. These results indicated that *APOE* ɛ4 status and methylation levels of *BIN1* promoter might contribute to early pathological changes of AD independently or jointly.

This was the first study to systematically explore the associations between methylation status of *BIN1* promoter in peripheral blood and preclinical AD in a large Han Chinese population. However, there were still some potential limitations in our study. Firstly, this was a cross-sectional study and results still need to be tested in larger longitudinal cohorts. Secondly, associations between methylation status of *BIN1* promoter and expression of *BIN1* gene in peripheral blood still need to be further explored in the future.

In summary, our study was the first to find a robust association of hypomethylation of *BIN1* promoter in peripheral blood with preclinical AD. Though the underlying mechanisms were not entirely clear, these robust results still provide new evidence for the involvement of *BIN1* methylation in the occurrence and development of AD. It is worth noting that our results suggest that these influences of *BIN1* hypomethylation on AD pathology might occur at a very early stage, which is more important for early intervention and prevention of AD. Furthermore, because methylation is more plastic than gene mutation, future studies in this direction will be more likely to find feasible and effective therapeutic targets for AD.

## Supplementary information

Additional file

## Data Availability

The datasets used and/or analysed during the current study are available from the corresponding author on reasonable request.
