# Supplementary material for: Association between methylation of BIN1 promoter in peripheral blood and preclinical Alzheimer’s disease
Source: Transl Psychiatry. 2021 Feb 2;11:89. doi: 10.1038/s41398-021-01218-9 (PMC7854626; doi:10.1038/s41398-021-01218-9)
Supplement: Supplementary file 1 — Additional file [file 41398_2021_1218_MOESM1_ESM.docx]

**Additional file 1. The information of quality control in our study**

**
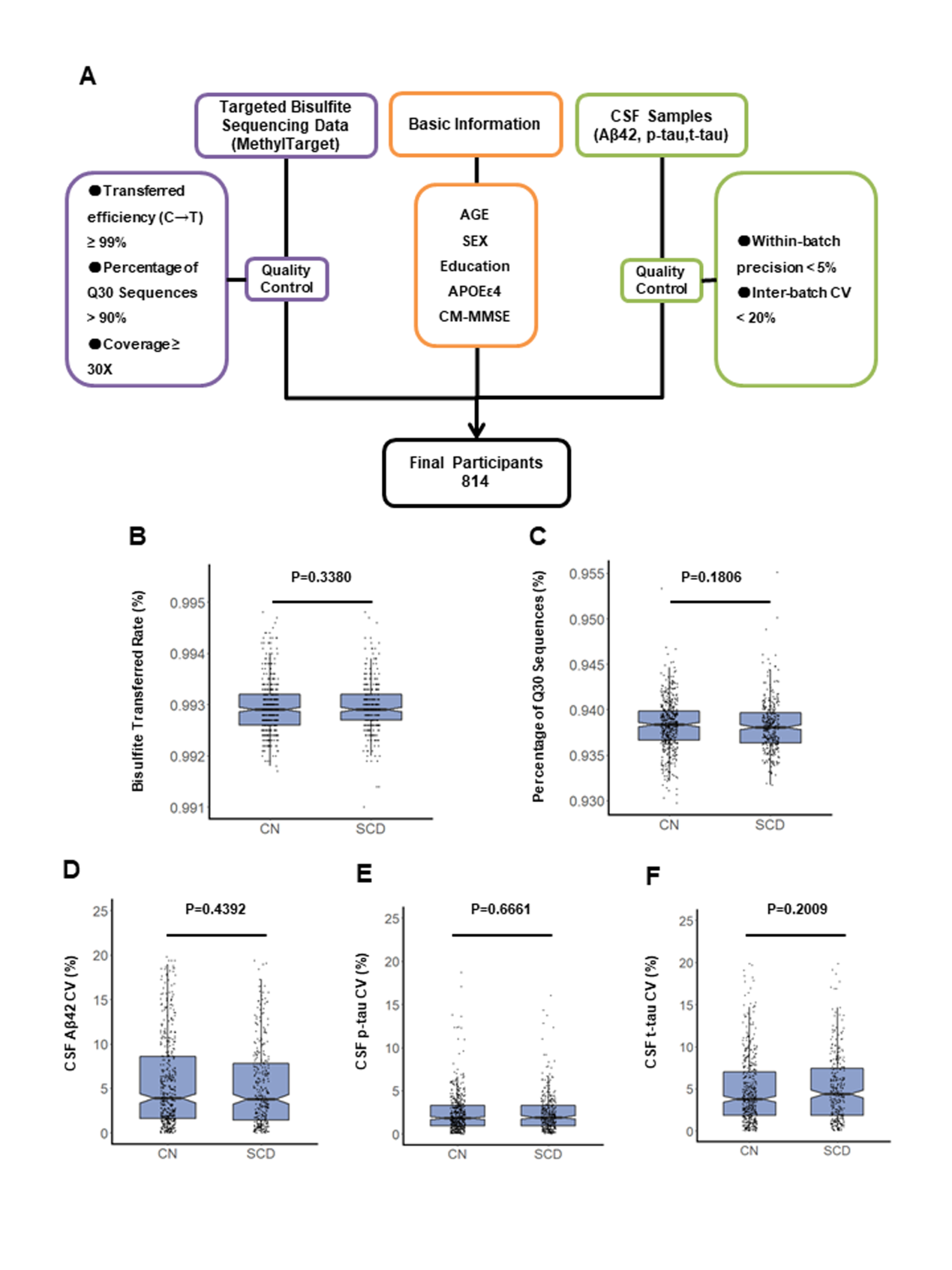
**

**Additional file 2. Associations of CSF Aβ42 levels with age and *APOE* ε4 status**

**
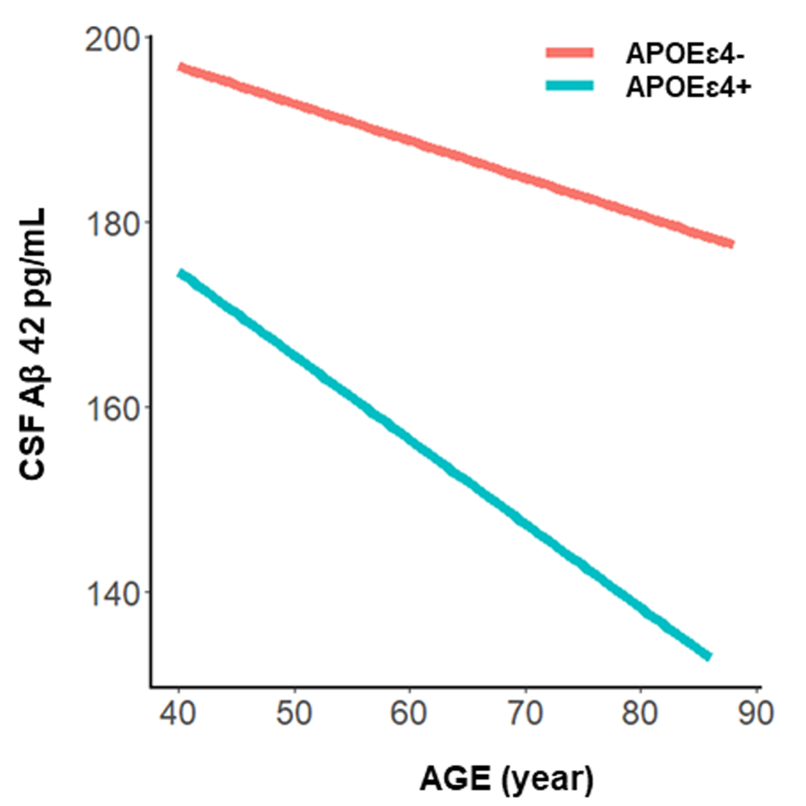
**

**Additional file 3. The information of six different regions on *BIN1* promoter**

| Region | mRNA | TSS | TES | Start | End | Length | Distance to TSS |
| --- | --- | --- | --- | --- | --- | --- | --- |
| **BIN1_01** | NM_001320642 | 127842126 | 127805598 | 127842075 | 127841830 | 246 | 51 |
| **BIN1_02** | NM_001320642 | 127842126 | 127805598 | 127865297 | 127865106 | 192 | -23171 |
| **BIN1_03** | NM_001320642 | 127842126 | 127805598 | 127864876 | 127865087 | 212 | -22750 |
| **BIN1_04** | NM_001320642 | 127842126 | 127805598 | 127864637 | 127864894 | 258 | -22511 |
| **BIN1_05** | NM_001320642 | 127842126 | 127805598 | 127864661 | 127864493 | 169 | -22535 |
| **BIN1_06** | NM_001320642 | 127842126 | 127805598 | 127864514 | 127864310 | 205 | -22388 |

**Abbreviations:** TSS: transcription start site; TES: transcription end site; Start: the start site of region; End: the end site of region; Length: the length of region; Distance to TSS: the distance between the region and TSS, “-” means that this site is upstream of the TSS.

**Additional file 4. Different methylation levels of CpG sites on BIN1_01** **region between CN and SCD subgroups**

**
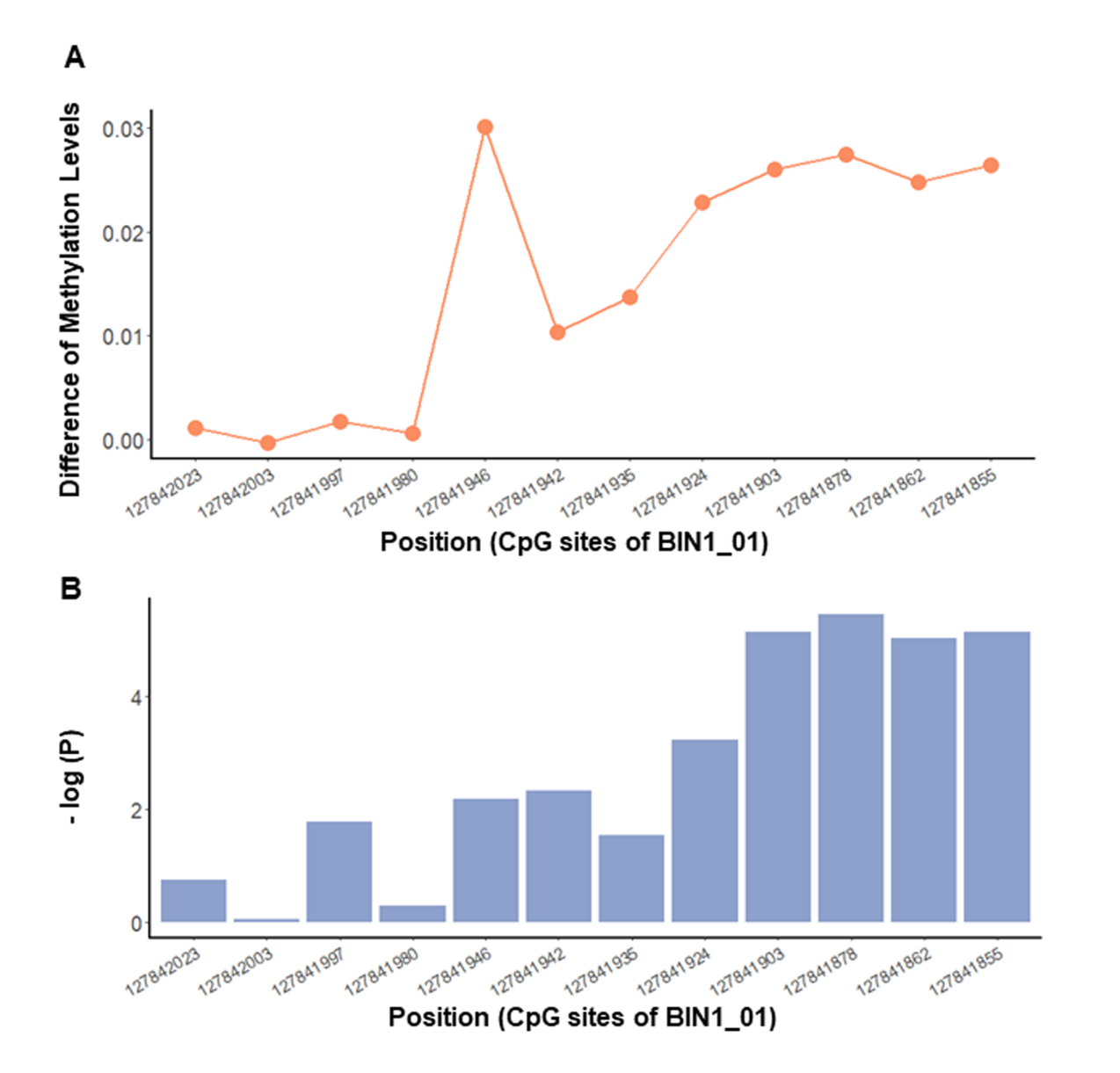
**

(A) The difference in methylation levels of CpG sites between the CN and SCD subgroups. (B) The p-value of the difference in methylation levels of CpG sites between the CN and SCD subgroups.

**Additional file 5. The area under the receiver operating characteristic curve of different *BIN1* promoter regions on diagnostic subgroups**

**
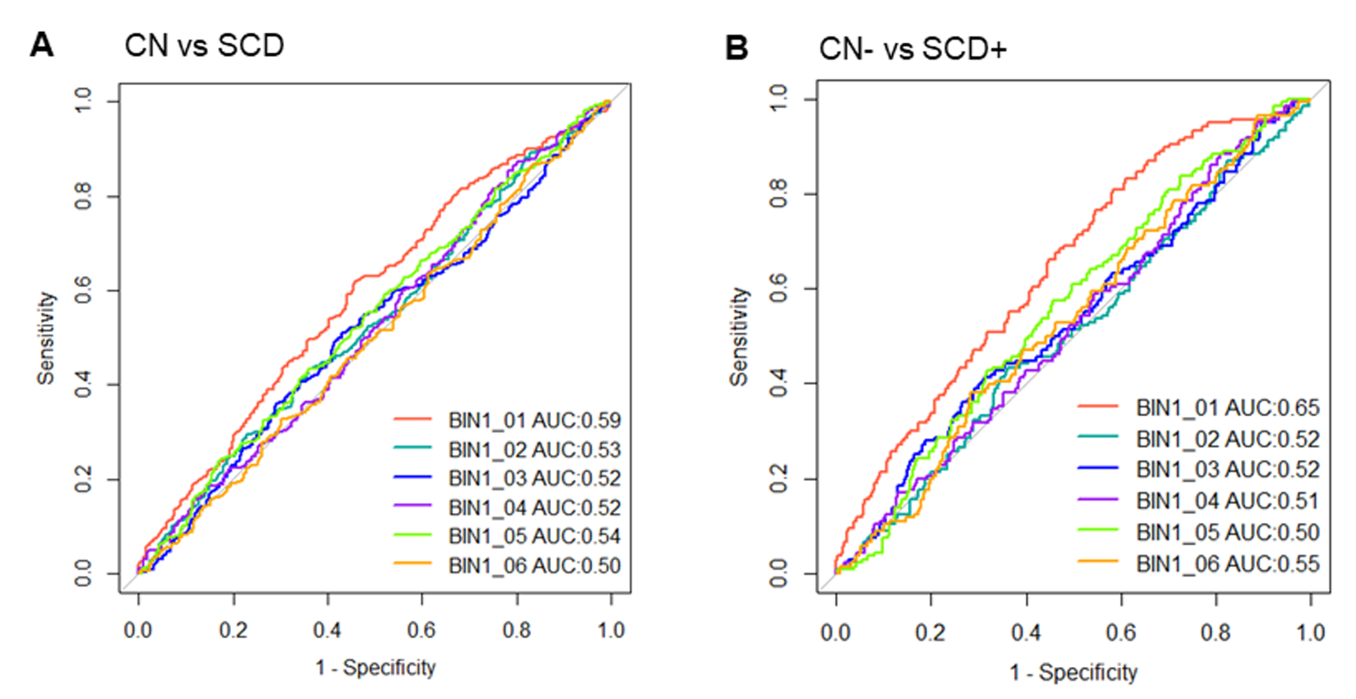
**

**Abbreviations:** CN, cognitively normal participants; SCD, participants with subjective cognitive decline; CN-: cognitively normal participants with negative CSF Aβ42 [A-]; SCD+: participants with subjective cognitive decline and positive CSF Aβ42 [A+]; BIN1: bridging integrator 1 gene promoter; BIN1_01~06: six different regions of bridging integrator 1 gene promoter; AUC: area under the receiver operating characteristic curve

**Additional file 6. Associations between methylation levels of different regions (BIN1_02~06) on *BIN1* promoter and CSF biomarkers in total and** **different diagnostic subgroups**

| **Variable** | **Total** | |  | **CN** | |  | **SCD** | |
| --- | --- | --- | --- | --- | --- | --- | --- | --- |
|  | **β** | **P** |  | **β** | **P** |  | **β** | **P** |
| **BIN1_02** | | | | | | | | |
| **CSF Aβ42** | -5.9708 | 0.5263 |  | -6.1117 | 0.6059 |  | -8.5585 | 0.5900 |
| **CSF p-tau** | -6.2243 | 0.5060 |  | 7.8159 | 0.4990 |  | -26.3166 | 0.1069 |
| **CSF t-tau** | -11.3100 | 0.2180 |  | -1.3544 | 0.9050 |  | -26.8625 | 0.0959 |
| **CSF p-tau/Aβ42** | 2.5601 | 0.7854 |  | 11.1946 | 0.3427 |  | -6.9732 | 0.6642 |
| **CSF t-tau/Aβ42** | -4.6082 | 0.6217 |  | 4.4842 | 0.7020 |  | -16.2072 | 0.3172 |
| **BIN1_03** | | | | | | | | |
| **CSF Aβ42** | -43.2108 | 0.0607 |  | -36.6000 | 0.2149 |  | -55.5927 | 0.1350 |
| **CSF p-tau** | 22.5087 | 0.3250 |  | 14.6871 | 0.6100 |  | 36.0960 | 0.3447 |
| **CSF t-tau** | 13.8611 | 0.5380 |  | 10.5923 | 0.7090 |  | 21.8098 | 0.5626 |
| **CSF p-tau/Aβ42** | 38.2656 | 0.0682 |  | 42.4929 | 0.1482 |  | 73.2338 | 0.0506 |
| **CSF t-tau/Aβ42** | 43.2066 | 0.0531 |  | 38.7837 | 0.1846 |  | 61.4922 | 0.1033 |
| **BIN1_04** | | | | | | | | |
| **CSF Aβ42** | -5.0296 | 0.8715 |  | -15.9300 | 0.6971 |  | 26.6407 | 0.5780 |
| **CSF p-tau** | 22.9991 | 0.4560 |  | 9.5980 | 0.8101 |  | 37.0465 | 0.4512 |
| **CSF t-tau** | 11.0524 | 0.7160 |  | -25.7165 | 0.5130 |  | 61.9096 | 0.2010 |
| **CSF p-tau/Aβ42** | 15.6826 | 0.6133 |  | 17.3932 | 0.6697 |  | -3.4469 | 0.9431 |
| **CSF t-tau/Aβ42** | 14.0100 | 0.6494 |  | -0.1225 | 0.9976 |  | 27.4413 | 0.5722 |
| **BIN1_05** | | | | | | | | |
| **CSF Aβ42** | 36.3500 | 0.0880 |  | 59.9037 | 0.0520 |  | 34.3078 | 0.4760 |
| **CSF p-tau** | -7.7259 | 0.7591 |  | -7.4290 | 0.8005 |  | -4.6112 | 0.9258 |
| **CSF t-tau** | 11.0342 | 0.6560 |  | 7.9177 | 0.7843 |  | 20.6505 | 0.6721 |
| **CSF p-tau/Aβ42** | -50.2937 | 0.0542 |  | -49.8118 | 0.0676 |  | -36.0117 | 0.4590 |
| **CSF t-tau/Aβ42** | -49.4800 | 0.0590 |  | -55.5172 | 0.0555 |  | -10.6519 | 0.8278 |
| **BIN1_06** | | | | | | | | |
| **CSF Aβ42** | -26.5662 | 0.1972 |  | -30.4200 | 0.2256 |  | -7.5949 | 0.8360 |
| **CSF p-tau** | -8.3536 | 0.6820 |  | 7.9379 | 0.7450 |  | -37.4684 | 0.3201 |
| **CSF t-tau** | 0.1261 | 0.9950 |  | 11.6334 | 0.6280 |  | -21.8933 | 0.5564 |
| **CSF p-tau/Aβ42** | 19.1767 | 0.3510 |  | 31.2959 | 0.2106 |  | -14.9813 | 0.6858 |
| **CSF t-tau/Aβ42** | 17.8983 | 0.3809 |  | 31.6447 | 0.2025 |  | -15.6669 | 0.6747 |

**Abbreviations:** CN, cognitively normal participants; SCD, participants with subjective cognitive decline; CSF, cerebrospinal fluid; Aβ, amyloid-β; p-tau, phosphorylated tau protein; t-tau, total tau protein; BIN1_02~06: five different regions of bridging integrator 1 gene promoter

Multiple linear regression models were used to test the associations between methylation levels of *BIN1* promoter and CSF biomarkers, adjusting for age, gender, education and *APOE* ɛ4 status.

**Additional file 7. Associations of SNPs with preclinical AD susceptibility, CSF biomarkers or methylation levels of *BIN1* promoter**

| **Model** | **rs58402148** | |  | **rs17014923** | |
| --- | --- | --- | --- | --- | --- |
|  | **Estimate** | **P** |  | **Estimate** | **P** |
| **Diagnose** |  |  |  |  |  |
| **CN VS SCD** | -0.3239 | 0.0568 |  | -0.2324 | 0.2189 |
| **CN- VS SCD+** | -0.3304 | 0.1711 |  | -0.4107 | 0.1338 |
| **CSF Biomarkers** |  |  |  |  |  |
| **CSF Aβ42** | -0.0033 | 0.8242 |  | -0.0015 | 0.9289 |
| **CSF p-tau** | 0.0013 | 0.8600 |  | -0.0135 | 0.1130 |
| **CSF t-tau** | 0.0042 | 0.7350 |  | -0.0031 | 0.8230 |
| **CSF p-tau/Aβ42** | 0.0046 | 0.7541 |  | -0.0120 | 0.4732 |
| **CSF t-tau/Aβ42** | 0.0075 | 0.6463 |  | -0.0016 | 0.9295 |
| **Methylation Levels** |  |  |  |  |  |
| **BIN1** | 0.0012 | **<0.0001** |  | 0.0004 | **<0.0001** |
| **BIN1_01** | 0.0830 | **<0.0001** |  | 0.0281 | **<0.0001** |

**Abbreviations:** CN, cognitively normal participants; SCD, participants with subjective cognitive decline; CN-: cognitively normal participants with negative CSF Aβ42 [A-]; SCD+: participants with subjective cognitive decline and positive CSF Aβ42 [A+]; BIN1: bridging integrator 1 gene promoter; CSF, cerebrospinal fluid; Aβ, amyloid-β; p-tau, phosphorylated tau protein; t-tau, total tau protein; BIN1: bridging integrator 1 gene promoter; BIN1_01: 01 region of bridging integrator 1 gene promoter

**Additional file 8. Associations between methylation levels of *BIN1* promoter and CSF biomarkers after adjusting peripheral blood cell composition and storage time.**

| **Variable** | **Total** | |  | **CN** | |  | **SCD** | |
| --- | --- | --- | --- | --- | --- | --- | --- | --- |
|  | **β** | **P** |  | **β** | **P** |  | **β** | **P** |
| **BIN1** | | | | | | | | |
| **CSF Aβ42** | 25.8183 | **0.0006** |  | 15.1877 | 0.1379 |  | 41.8781 | **0.0002** |
| **CSF p-tau** | -13.5768 | 0.0726 |  | 1.8112 | 0.8565 |  | -25.9540 | **0.0269** |
| **CSF t-tau** | -12.4500 | 0.0927 |  | 4.1014 | 0.6777 |  | -25.7093 | **0.0270** |
| **CSF p-tau/Aβ42** | -33.0531 | **<0.0001** |  | -15.8037 | 0.1234 |  | -53.5567 | **<0.0001** |
| **CSF t-tau/Aβ42** | -32.3175 | **<0.0001** |  | -11.0996 | 0.2794 |  | -53.3546 | **<0.0001** |
| **BIN1_01** | | | | | | | | |
| **CSF Aβ42** | 2.9408 | **0.0003** |  | 1.8610 | 0.0936 |  | 4.5784 | **0.0002** |
| **CSF p-tau** | -1.5053 | 0.0676 |  | 0.3055 | 0.7785 |  | -3.0461 | **0.0173** |
| **CSF t-tau** | -1.3172 | 0.1020 |  | 0.5988 | 0.5758 |  | -2.9216 | **0.0212** |
| **CSF p-tau/Aβ42** | -3.7304 | **<0.0001** |  | -1.8648 | 0.0935 |  | -5.9778 | **<0.0001** |
| **CSF t-tau/Aβ42** | -3.6101 | **<0.0001** |  | -1.2905 | 0.2460 |  | -5.9485 | **<0.0001** |

**Abbreviations:** CN, cognitively normal participants; SCD, participants with subjective cognitive decline; CSF, cerebrospinal fluid; Aβ, amyloid-β; p-tau, phosphorylated tau protein; t-tau, total tau protein; BIN1: bridging integrator 1 gene promoter; BIN1_01: 01 region of bridging integrator 1 gene promoter

Bold indicated that the results were statistically significant.

Multiple linear regression models were used to test the associations between methylation levels of BIN1 promoter and CSF biomarkers, adjusting for age, gender, education, *APOE* ɛ4 status, two SNPs (rs58402148; rs17014923), peripheral blood cell composition (neutrophile granulocyte, lymphocyte, monocyte, eosinophilic granulocyte and basophilic granulocyte) and storage time.
